# HMGB1-TIM3-HO1: A New Pathway of Inflammation in Skin of SARS-CoV-2 Patients? A Retrospective Pilot Study

**DOI:** 10.3390/biom11081219

**Published:** 2021-08-16

**Authors:** Gerardo Cazzato, Anna Colagrande, Antonietta Cimmino, Gerolamo Cicco, Vincenza Sara Scarcella, Paola Tarantino, Lucia Lospalluti, Paolo Romita, Caterina Foti, Aurora Demarco, Sara Sablone, Pragnell Maria Victoria Candance, Sebastiano Cicco, Teresa Lettini, Giuseppe Ingravallo, Leonardo Resta

**Affiliations:** 1 Section of Pathology, Department of Emergency and Organ Transplantation (DETO), University of Bari “Aldo Moro”, 70124 Bari, Italy; anna.colagrande@gmail.com (A.C.); micasucci@inwind.it (A.C.); jerrycicco1@gmail.com (G.C.); vincenza.scarcella@policlinico.ba.it (V.S.S.); tarpa80@gmail.com (P.T.); mpragnellv@libero.it (P.M.V.C.); lettinit@yahoo.com (T.L.); leonardo.resta@uniba.it (L.R.); 2 Section of Dermatology, Department of Biomedical Sciences and Human Oncology, University of Bari “Aldo Moro”, 70124 Bari, Italy; l.lospalluti@gmail.com (L.L.); paolo.romita@uniba.it (P.R.); caterina.foti@uniba.it (C.F.); aurorademarco94@gmail.com (A.D.); 3 Section of Legal Medicine, Department of Interdisciplinary Medicine, Bari Policlinico Hospital, University of Bari, 70124 Bari, Italy; sarasabloneml@gmail.com; 4 Section of Internal Medicine “G. Baccelli”, Department of Biomedical Sciences and Human Oncology, University of Bari Medical School, 70124 Bari, Italy; sebastiano.cicco@uniba.it

**Keywords:** HMGB1, TIM-3, HO-1, SARS-CoV-2, skin

## Abstract

The SARS-CoV-2 pandemic has completely disrupted the health systems of the entire planet. From the earliest months, it became increasingly clear that in addition to affecting the upper airways and lungs, there were other organs that could be affected. Among these, the skin became a real “sentinel signal” to be able to even suspect COVID-19. *Background*: this study deals with a little-explored issue for now: the study of skin immunopathology in SARS-CoV-2 positive subjects ascertained using the most reliable methods available. *Methods*: we used skin biopsy samples from SARS-CoV-2 positive and negative patients, studying morphology (Hematoxylin-Eosin), T lymphocyte population (CD4 and CD8), three markers such as HMGB-1, TIM-3 and HO-1 by immunohistochemistry. *Results*: although the presence of the CD4 and CD8 T population did not differ statistically significantly, we found greater activation and release of HMGB-1 in skin samples from SARS-CoV-2 positive patients, greater immunolabeling for TIM-3 at the level of CD4 and CD8 and a reduced expression of Heme oxygenase 1. *Conclusions*: these results support the possibility that there is immune deregulation in SARS-CoV-2 positive patients who develop skin manifestations of various kinds.

## 1. Introduction

The SARS-CoV-2 pandemic has completely undermined entire global health and economic systems and by 18 June 2021, caused 177,108,695 confirmed cases and 3,840,223 deaths [1]. Countless different manifestations have been reported in the course of COVID-19, among which rashes have gained a place of some importance [2,3,4,5,6,7]. Different works have attempted to relate the severity of the skin manifestations with the possible outcome of the affected patient, but it is still debated today whether the skin manifestations can really be considered a “predictor” of the disease severity [8,9,10]. With the aim of exploring whether there is a causal link between immunopathology and the severity of skin manifestations, we conducted this work at our Histology and Microscopic Anatomopathology Operative Unit, studying eight cases of small skin biopsies of SARS-CoV-2 positive patients with skin manifestations. The samples were immunostained with three antibodies, HMGB1, TIM-3 and H-OX1, three markers involved both in innate/adaptive immunity and in the regulation of the immunological response (TIM-3 and HO-1), as well as in the proinflammatory and procytokine response (HMGB-1).

We present the results and conduct a brief discussion of the therapeutic perspectives in light of the results obtained.

### 1.1. Background

#### 1.1.1. HIGH MOBILITY GROUP BOX 1 (HMGB-1)

High mobility group box 1 (HMGB1) is a non-histone nuclear protein, a prototype of the so-called damage-associated molecular pattern (DAMPs) or alarmines, discovered almost four decades ago [11], that has a dual role. Within the nucleus, it binds DNA without sequence specificity and induces conformational changes that facilitate interaction between the double helix and multiple transcription factors, including p53, NF-kB, RAG 1/2 and steroid hormone receptors [12], involved in important biological functions such as regulation of the inflammatory cascade, cell differentiation and proliferation, as well as apoptosis [13]. When stress or cell damage ranging up to necrosis occurs, HMGB1 is released into the extracellular environment, exerting pro-inflammatory, immuno-regulatory and pro-tumor effects through autocrine and paracrine mechanisms. Although about 14 receptors that can bind HMGB1 have been identified, the most important and best characterized are the toll-like receptors (TLR and, in particular, TLR4) and RAGE (receptor for advanced glycation end-products) [14]. At the immune level, HMGB1 promotes the activation and maturation of antigen-presenting cells (APCs) and dendritic cells (DCs) in particular, facilitating their migration to the lymph node stations. Here, DCs activated by HMGB1 following binding to the RAGE receptor, a transmembrane protein belonging to the immunoglobulin superfamily, induce the proliferation and clonal expansion of alloreactive T lymphocytes, and a strong T helper 1 (Th 1) polarization, as evidenced by the production of IL-2 and IFN-γ [12,15]. HMGB1 therefore exerts its effects mainly on innate immunity cells (DCs, monocytes/macrophages), but there is also evidence of its influence on lymphoid cells on both T helper and T regulators, on B lymphocytes, and on natural cells. killer (NK), by binding to RAGE, TLR2/4 and TIM-3, RAGE and TLR2/4/9, and TRL2/4 and TIM-3, respectively [15]. By binding with TLR4 it suppresses the proliferation of T lymphocytes and the production of IFNγ, indicating that the co-stimulatory signal of TCR is abrogated by HMGB1 [15]. HMGB1 can therefore be seen as a molecule acting as a bridge between innate immunity and acquired immunity.

#### 1.1.2. T CELL IMMUNOGLOBULIN and MUCIN DOMAIN 3 (TIM-3)

T cell immunoglobulin and mucin domain 3 (TIM-3) is a member of the TIM gene family, located on chromosome 5q33.2, which includes type I membrane glycoproteins that share a similar structure; a variable immunoglobulin domain (IgV) with a stem consisting of glycosylated mucins of different lengths in the extracellular portion, a single transmembrane domain and an intracytoplasmic tail comprising five highly conserved tyrosine residues, with essential functions [16]. It was first identified about two decades ago by Monney and collaborators, who demonstrated its presence on differentiated Th1 lymphocytes, correlating it with autoimmune diseases [16,17]. Tim-3 is expressed not only on Th1 lymphocytes, but also on Th 17, cytotoxic CD8+ lymphocytes, NK cells, and myeloid lineage cells [16,18]. It is among the so-called immunological checkpoints, or co-inhibitory receptors, the prototypes of which are CTLA-4 and PD-1. Some tumors have the ability to produce inhibitory ligands, which bind to these receptor checkpoints on tumor-specific immune cells, inhibiting their functions and allowing the tumor to evade immune surveillance [19]. There are four ligands capable of interacting with the IgV domain of TIM-3: HMGB1, the aforementioned nuclear protein released by necrotic cells; galectin-9 (Gal-9), a plasma and nuclear glycoprotein involved in signal transduction processes and in numerous aspects of tumor progression [20]; the adhesion molecule of carcinoembryonic antigen cells (carcinoembryonic antigen cell adhesion molecule or Ceacam-1), expressed on activated T lymphocytes [21]; phosphatidyl serine (PtdSer) exposed on the membranes of apoptotic cells [21]. These ligands can be released from necrotic cells, tumor cells, or be present on the surface of T lymphocytes [16]. The binding of TIM-3 to its ligands, overall, exerts an inhibitory role on T lymphocytes, suppressing Th1 and Th17 responses [17,22], but also reducing the proliferation and production of cytokines by CD8 + lymphocytes [17]. Specifically, Gal-9, the first TIM-3 ligand to be described, specifically recognizes a glucidic motif on TIM-3 IgV in the antigen-specific CD4 + Th1, inducing apoptosis. Moreover, its expression on Treg lymphocytes is critical for the suppression of TIM-3 + effector T lymphocytes function [22]. HMGB1 essentially binds to TIM-3 expressed on DCs, inhibiting its activation [17], but it has been postulated that the HMGB1/TIM-3 complex can also directly downregulate cellular T responses, binding CD8 + Tregs which, in turn, suppress the proliferation of effector T cells [16,23]. Therefore, HMGB1 may trigger TIM-3-dependent inhibitory pathways both in innate immunity cells and in T cells [16]. Ceacam-1 is a negative regulator of the T lymphocyte response, and PtdSer facilitates the presentation of the antigen to DCs and the clearance of apoptotic bodies, maintaining immunological tolerance [17,24,25].

#### 1.1.3. HEME OXYGENASE-1

Heme oxygenase-1 (HO-1) is a 32 kDa inducible microsomal enzyme that catalyzes the first reaction, a limiting step, of the heme degradation enzymatic pathway, with the production of equimolar quantities of carbon monoxide (CO), ferrous ion (Fe^2+^), a potentially toxic product from which free radicals can be derived, and biliverdin [26]. The enzymatic activity of HO-1 results in a decrease in oxidative stress, which results in an attenuation of the inflammatory response, and a reduced rate of apoptosis. This is achieved both due to the removal of heme, a powerful pro-oxidant and pro-inflammatory agent, but also to the generation of biologically active products, of which CO is the most important cell mediator. In fact, it has signal functions that resemble those of nitric oxide (NO): it induces soluble guanylate cyclase, consequently inhibiting platelet aggregation, causing a decrease in leukocyte adhesion and reducing apoptosis of endothelial cells. In addition, CO exerts anti-inflammatory effects through the inhibition of TNF, IL-1β and the inflammatory protein of macrophages-1β (MIP-1β), or through the upregulation of IL-10 [26,27]. Biliverdin is, together with bilirubin, an inhibitor of the complement cascade and a powerful antioxidant, capable of attenuating the inflammatory response and the peroxidation of lipid membranes and proteins through a scavenger action on peroxyl radicals [28]. There are multiple stimuli capable of inducing HO-1 expression in response to cellular stress and oxidative stimuli: UV radiation, reactive oxygen species (ROS), its own heme substrate, inflammatory cytokines, prostaglandins, heavy metals, and hypoxia [26]. A few cells constitutively express HO-1, including CD4 +/CD25 + regulatory T lymphocytes [26].

### 1.2. Rational

We decided to study the immunopathology of skin manifestations from SARS-CoV-2 more deeply; for this reason, we studied the T-arm using immunomarkers for CD4 and CD8 that could define if there were quantitative differences between skin samples from SARS-CoV-2 positive and control negative patients. Once this was determined, we investigated whether there were differential expressions of molecules released in inflammatory situations (such as HMGB-1), whether the T lymphocytes present were deregulated or “exhausted” in their function by immunolabeling for TIM-3 and, finally, to evaluate whether an enzyme with clearly “antioxidant” activity such as HO-1, could be downregulated in patients testing positive for the virus.

## 2. Materials and Methods

### 2.1. Procedure

A retrospective study was conducted of 8 cases of patients with skin manifestations from SARS-CoV-2, subjected to 3.5/4 mm punch biopsies. The patients had been referred both from the Dermatology and Venereology Unit of Bari University Polyclinic, and from other private professionals. In all cases, the patient’s informed consent was obtained. Preparation of the slides and the histological diagnosis, as well as the reading and immunohistochemical investigations, were conducted at the U.O.C. of University Pathological Anatomy of Bari Polyclinic. The total cohort consisted of 2 groups; 8 patients with a SARS-CoV-2 positive molecular swab [GeneXpert Dx Xpress SARS-CoV-2 RT-PCR assay (Cepheid) with sensitivity and specificity of 100% (87/87 samples) and 100% (30/30 samples), with a limit of detection of 250 copies/mL or 0.0100 plaque-forming units per milliliter] with at least one skin biopsy performed in correspondence of the affected skin region, and 8 patients (control group) belonging to the control group, with a negative molecular swab for SARS-CoV-2. The first group included 5 F and 3 M, aged between 16 and 63 years; the topographic distribution of the skin lesions in almost all cases was on the upper limbs, lower limbs and trunk. Five patients presented mild/moderate symptoms with cough, myalgia and arthralgia; the remaining 3 were completely asymptomatic. Three patients presented a maculo-papular eruption at the level of the regions of the trunk and of the upper and lower limbs; 2 patients presented “childblain-like” lesions in the lower limbs (mainly malleolar and perimalleolar region) and 3 patients presented with varicelliform skin rash.

The control group consisted of 6 F and 2 M, aged between 18 and 71 years, who had undergone skin excision for aesthetic–functional reasons, having no skin or other pathology worthy of note in their history. A detailed history and clinical–pathological reconstruction were not significant for any pathology.

**Figure 1:**
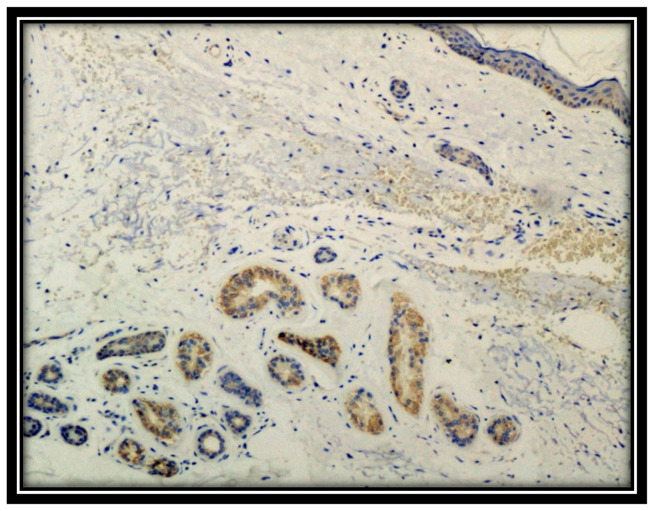
Immunostaining for S1 spike protein of SARS-CoV-2. Note the positivity at the level of eccrine sweat glands (Immunohistochemistry, Original Magnification: 10×).

The punch biopsy samples for histological examination were fixed in 10% neutral buffered formalin, dehydrated, and embedded in paraffin. From these paraffinized blocks, 5 µm sections were obtained, dewaxed, hydrated and subjected to routine staining with Hematoxylin-Eosin and, subsequently, to immunohistochemical investigations using antibodies for the following markers: CD4, CD8, HMGB1, TIM-3 and Heme oxygenase-1 (HO-1). Furthermore, all the biopsies of the patients in question were subjected to immunostaining for SARS-CoV-2: the patients with a positive molecular swab were positive for the Spike protein in the skin, the signal being mainly localized at the level of the eccrine sweat glands (anti-SARS-CoV-2 spike S1 glycoprotein monoclonal antibody, Thermo Fisher (Rochester, NY, USA), Rabbit, at pH 6, diluted 1:800, and antigenic unmasking heat-induced citrate buffer epitope retrieval) (Figure 1). Biopsies from patients with a negative SARS-CoV-2 molecular swab were also negative for immunohistochemistry (IHC). All skin biopsies were tested with RT-PCR (TaqMan 2019-nCoV Assay Kit v1, Thermo Fisher Scientific (USA), which targets three different viral genomic regions (ORFab1, S protein, and N protein), which was positive in the 8 cases of established positivity albeit with a low number of copies.

The data relating to each clone of antibody used are shown in Table 1. Reading of the immunohistochemical reactions was performed by evaluating the cell density for the CD4 and CD8 markers, counting positive cells in 10 high magnification fields (HPF) at 400× for each clinical case. A Reichert Polyvar 2 microscope with JTV digital camera and Trinitron monitor, Sony, was used. Each field was analyzed at a magnification of 400×; the size of each field was 140 microns in length by 110 microns in width, the total field width being 15,400 square microns. Evaluation of the expression of HMGB1, TIM-3 and Heme-oxygenase-1 was carried out by highlighting the chromogen signal on the plasma membrane, in the nucleus, in the cytoplasm, or in the extracellular medium of the samples examined. For HMGB1, TIM-3 and HO-1 a score was assigned, given by the sum of a score related to the different degrees of staining intensity (degree 0 = no staining; degree 1 = weak; degree 2 = moderate; degree 3 = intense staining) and a score related to the percentage of extension (score 0: <1%; score 1: 1–25%; score 2: 26–50%; score 3: 51–74%; score 4: ≥75%). The final score was considered high if the sum of the two scores exceeded 3 [29,30,31].

### 2.2. Statistical Analysis

From the values obtained in the 10 fields at 400× (HPF) for each individual patient, the mean and standard deviation were calculated. The data were analyzed by Kolmogorov–Smirnov test to check for a normal distribution of the values. In the case of *p* > 0.05, the values were considered normally distributed and therefore subjected to parametric testing. Otherwise, non-parametric testing was used for statistical analysis. We made comparisons between the means of the individual groups and then we compared the two groups under study. The TIM-3, HMGB1 and OH-1 evaluations were non-parametric and analysis of the means was carried out using the Mann–Whitney test. The values of CD4, CD8, being parametric, were analyzed by Student’s *t*-test for means. A *p*-value of less than 0.05 (*p* < 0.05) was considered significant. All statistical analyses were performed using the Prism 9.2.0 program, GraphPad Software, La Jolla (San Diego, CA, USA).

## 3. Results

**Figure 2:**
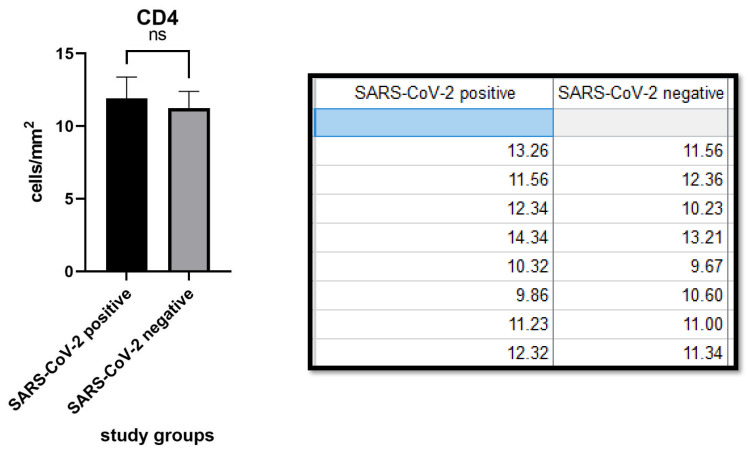
Table and graph relating to the density of CD4+ lymphocytes in the samples of the two groups. Note that there was no statistically significant difference (*p* value 0.3380). ns notes not significant.

CD4 expression was 11.90 ± 1.48 (cells/mm^2^) in the SARS-CoV-2 positive patients group and 11.25 ± 1.45 (cells/mm^2^) in the negative patients group. The difference between the two groups was not statistically significant (*p* value: 0.3380, >0.05). (Figure 2).

**Figure 3:**
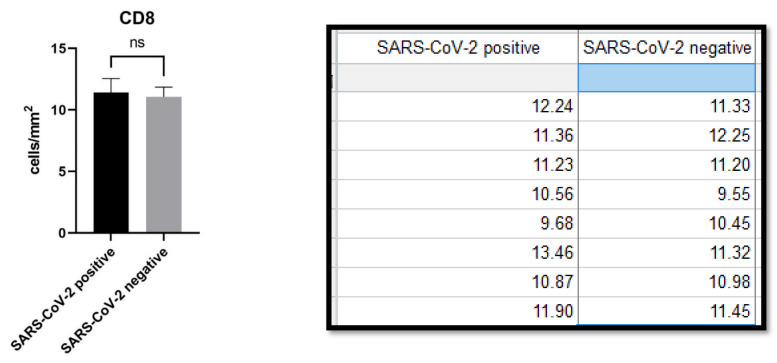
Table and graph relating to the density of CD8+ lymphocytes in the samples of the two groups of studies. Note that, also in this case, there was no statistically significant difference (*p* value: 0.4932, >0.05). ns notes not significant.

CD8 expression was 11.41 ± 1.145 (cells/mm^2^) in the SARS-CoV-2 positive group versus 11.07 ± 0.7916 (cells/mm^2^) in the negative group. There was no difference between the first and second groups (*p* value: 0.4932, >0.05). (Figure 3).

**Figure 4:**
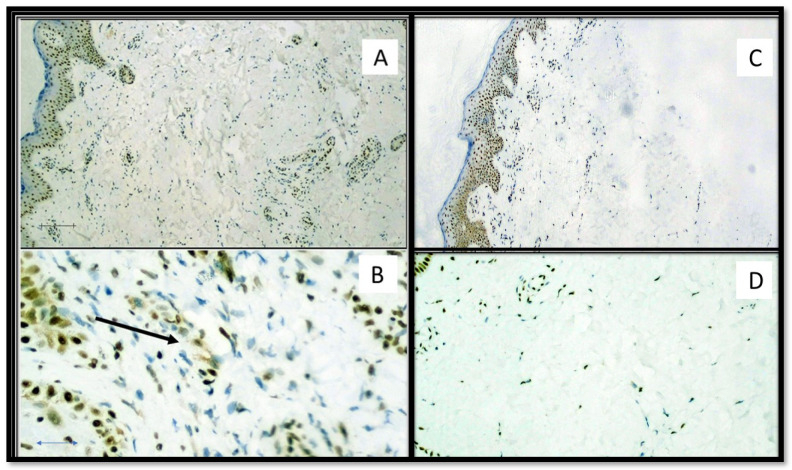
(**A**) Immunostaining for HMGB1 of a patient positive for SARS-CoV-2: note the internal positive control of normal epidermal keratinocytes and positivity in the cells constituting the excretory portion of the eccrine sweat glands that were strongly positive for anti-S1 spike protein immunostaining by SARS-CoV-2 (Immunohistochemistry, Original Magnification: 40×). (**B**) Detail of extracellular release of HMGB1 (Immunohistochemistry, Original Magnification: 200×). (**C**) Immunostaining for HMGB1 of a patient negative for SARS-CoV-2: note the internal positive control and substantial negativity in the cells of superficial dermis (Immunohistochemistry, Original Magnification: 40×). (**D**) Detail of negativity for HMGB1 (Immunohistochemitry, Original Magnification: 100×).

**Figure 5:**
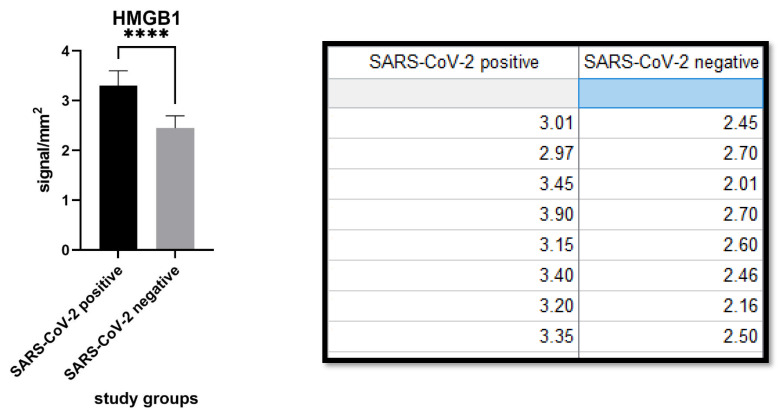
Different levels of HMGB1 immuno-expression in the two study populations. Note that there was statistically significant difference (*p* value < 0.0001, <0.05). The signal was calculated in a semi-quantitative way as reported in the paragraph “Material and Methods”. **** statistic significative.

HMGB1 expression was 3.304 ± 0.2977 (cells/mm^2^) in the SARS-CoV-2 positive patients group and 2.448 ± 0.2472 (cells/mm^2^) in the SARS-CoV-2 negative patients group. The difference was statistically significant (*p* value: <0.0001). (Figure 4 and Figure 5).

**Figure 6:**
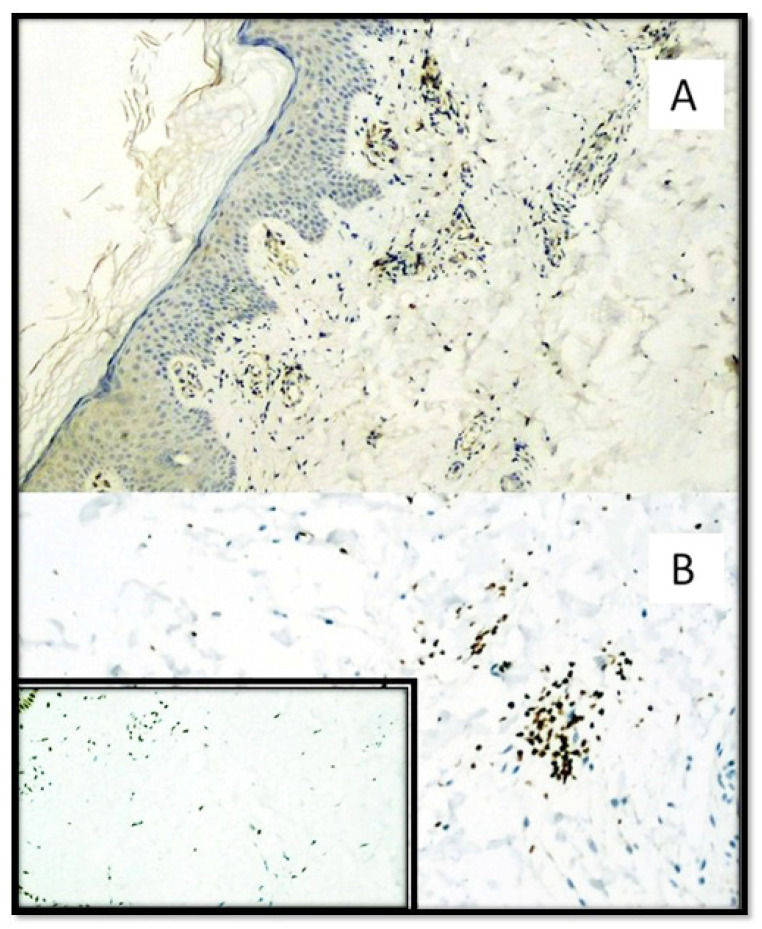
(**A**) TIM-3 immunostaining of SARS-CoV-2 positive patient skin sample by both molecular swab and S1-spike protein immunohistochemistry. Note the presence of predominantly subepithelial T lymphocyte clusters strongly positive for TIM-3. (Immunohistochemistry, Original Magnification: 100×). (**B**) Detail of a cluster of TIM-3 positive T lymphocytes (Immunohistochemistry, Original Magnification: 400×). **Box**: negative control for TIM-3 in skin biopsy from patient negative for SARS-CoV-2 (Immunohistochemistry, Original Magnificaton: 40×).

**Figure 7:**
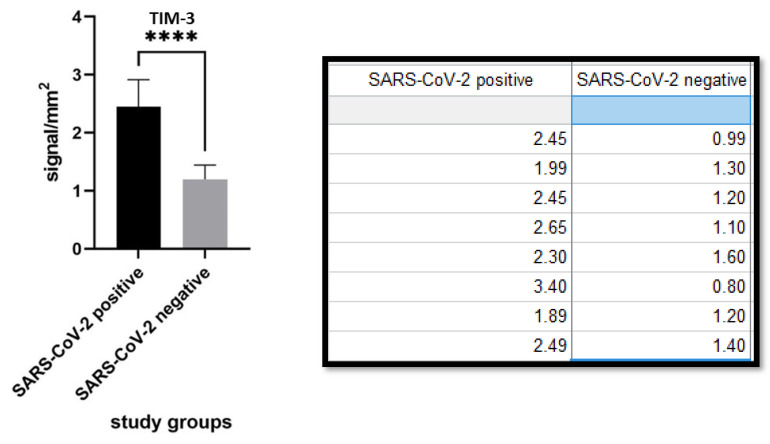
Different levels of immunoexpression also for TIM-3 in the two populations: note that, with the same lymphocyte population (CD4 and CD8 not statistically different), the lymphocytes present in the study arm of SARS-CoV-2 positive patients were immunolabeled for TIM-3 more than in the second group (*p* value: <0.0001). **** statistic significative.

TIM-3 expression was 2.453 ± 0.4616 (cells/mm^2^) in the SARS-CoV-2 positive patients group and 1.199 ± 0.2461 (cells/mm^2^) in the SARS-CoV-2 negative patients group. The difference was statistically significant (*p* value < 0.0001). (Figure 6 and Figure 7).

**Figure 8:**
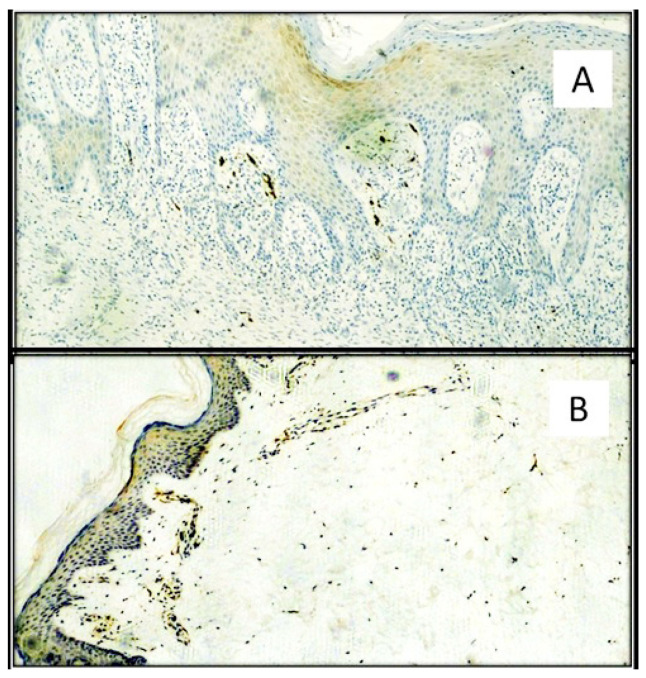
(**A**) Immunostaining for HO-1: note the positivity of a few inflammatory cells available mainly subepithelial in the skin of a patient positive for SARS-CoV-2 (Immunohistochemistry, Original Magnification: 40×). The light brown color is linked to “background noise” of the immunohistochemical reaction. (**B**) Photomicroghaph of skin biopsy of a patient negative for SARS-CoV-2 with higher expression of HO-1 in the epidermis and of inflammatory cells in subepithelial disposition (Immunohistochemistry, Original Magnification: 100×).

**Figure 9:**
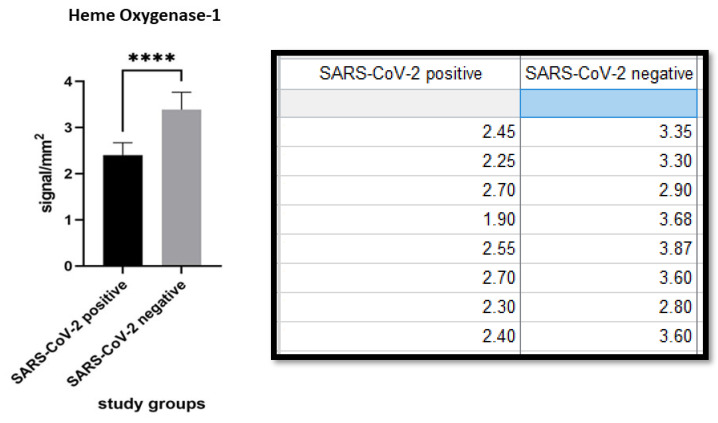
Comparison of immunoexpression for HO-1 between the two populations. Note the statistically significant difference (*p* value < 0.0001). **** statistic significative.

Heme Oxygenase 1 expression was 2.406 ± 0.2638 (cells/mm^2^) in the SARS-CoV-2 positive group and 3.388 ± 0.3781 (cells/mm^2^) in the SARS-CoV-2 negative patients. The difference was statistically significant (*p* value < 0.0001). (Figure 8 and Figure 9).

## 4. Discussion

The SARS-CoV-2 pandemic has triggered a major medical emergency around the world [1,32]. After more than a year and a half of the pandemic, many different etiopathogenic mechanisms have been studied to explain the multiple clinical outcomes of affected subjects [32,33]. Several immunopathologic mechanisms have been postulated, potentially responsible for the different skin manifestations associated with SARS-CoV-2 [34]. In this scientific landscape, our contribution aimed to explore whether or not there could be a link between these three markers and the SARS-CoV-2 manifestations. Some authors [34,35] have already highlighted the close link between HMGB1 release and exaggerated host inflammatory response in SARS-CoV-2 positive patients; this mechanism is one of the main causes of lung damage and subsequent mortality in many severe inflammatory lung conditions. The HMGB1-RAGE axis is expected to be overexcited in COVID-19 as necrotic respiratory epithelial cells contribute large amounts of extracellular HMGB1 and its cognate RAGE receptor is constitutively abundantly expressed in the lungs. Once triggered, inflammation would be intensified via HMGB1 secretion from innate immunity cells, causing further upregulation of RAGE and TLR4. Specifically, Andersson et al. focused attention on the fact that HMGB1 is a molecule that binds to DNA and RNA and HMGB1 has been shown to function as a gene delivery agent. There is a risk that HMGB1 could bind to viral RNA and carry it to the cytosol via the lysosomal RAGE pathway. This would imply that there may be an additional pathway beyond the angiotensin 2 conversion enzyme receptors that allow replication of the intracellular virus [35,36]. Street, in a recent paper, highlighted the need to assay HMGB1 in serum samples from COVID-19 patients who have been affected differently and who, as a rule, receive different treatment. This could, according to the author, clarify whether HMGB1 could be a marker of poor prognosis and a potential target for treatment [37,38]. In this study, we observed that the expression of HMGB1, TIM-3 and HO-1 was different in the two groups studied. In particular, HMGB1 was more strongly expressed both intracellularly and extracellularly (probably acting as damage-associated molecular patterns (DAMPs)) in the affected patients than in the control cases. Conversely, although there was no statistical difference in the amount of CD4 and CD8 lymphocytes between the two cohorts under examination, the immunoexpression of TIM-3 was greater in the SARS-CoV-2 positive group, almost as if most of the lymphocytes present in the skin were deregulated and/or inadequate to perform their functions. Finally, HO-1 was more strongly expressed in the cutis controls than in the SARS-CoV-2 positive group. We cannot make firm hypotheses but can postulate a reduced tolerance to oxidative stress (reduction in HO-1), probably due to the underlying inflammatory state induced by SARS-CoV-2.

In light of this, considering the potential link that HMGB1 is able to forge with TIM-3 [39] we hypothesize that higher levels of HMGB1 in patients with skin manifestations from SARS-CoV-2 are able to bind TIM-3 more, contributing to an immune dysregulation that manifests itself differently depending on other individual factors.

There are currently no reports in the literature about HMGB1, TIM-3 and HO-1 and their potential correlation in patients with skin manifestations from SARS-CoV-2. To our knowledge, this is the first study of this issue, although it has been shown that levels of HMGB1, for example, increased even before the development of cutaneous-origin malignancies, such as primary cutaneous lymphomas [38] These data are very interesting and may support future studies aiming to probe the possibility of a direct link between these results and more serious clinical outcomes.

## 5. Conclusions

Our study attempts to investigate, in greater detail, the mechanisms of immunopathology in SARS-CoV-2 positive patients who develop skin manifestations of various kinds. To do this, we analyzed three markers with immunohistochemistry that are affected at various different etiopathogenic moments: HMGB1 which promotes inflammation by acting as a phlogogenic cytokine; TIM-3 which is associated with immune dysregulation and/or functional “exhaustion” of T lymphocytes; and Heme oxygenase 1 which, as an emerging enzyme with antioxidant activity, is somehow reduced as an expression in the cutaneous microenvironment of the patient object of our study.

New studies and new research experiences with a greater number of patients are warranted to elucidate immune-mediated damage mechanisms in the skin and to understand if, and to what extent, this potential pathway can be exploited as a therapeutic target.

## Figures and Tables

**Table 1:** Summary of the salient features of the immunomarkers used in this study.

Name	Production	Code	Technique	Dilution	Pretreatment
CD4	Cell Marque lifescreen Ltd.	CMC1043101	HRP-DAB	1:100	EDTA
CD8	Cell Marque lifescreen Ltd.	CMC1083100	HRP-DAB	1:500	EDTA
HMGB1 IgG Rabbit polyclonal	Abcam	Ab18256	HRP-DAB	1:1000	Citrate buffer
TIM-3 IgG Rabbit Polyclonal	GeneTex Inc.	GTX 54117	HRP-DAB	1:100	Citrate buffer
HO-1 IgG Rabbit polyclonal	GeneTex Inc.	GTX101147	HRP-DAB	1:500	Citrate buffer
SARS-CoV-2, Anti-S1 Spike Protein	Thermo Fisher (USA)	MA5-36247	HRP-DAB	1:1000	EDTA

## Data Availability

Cicco S, Cicco G, Racanelli V, Vacca A. Neutrophil Extracellular Traps (NETs) and Damage-Associated Molecular Patterns (DAMPs): Two Potential Targets for COVID-19 Treatment. Mediators Inflamm. 2020 Jul 16;2020:7527953.
